# Temperamental factors predict long-term modifications of eating disorders after treatment

**DOI:** 10.1186/1471-244X-13-288

**Published:** 2013-11-07

**Authors:** Cristina Segura-García, Dora Chiodo, Flora Sinopoli, Pasquale De Fazio

**Affiliations:** 1 Department of Health Sciences, Chair of Psychiatry, University Magna Graecia, Catanzaro, Italy; 2 Ambulatory for Clinical Research and Treatment of Eating Disorders, University Hospital Mater Domini, Catanzaro, Italy

**Keywords:** Anorexia nervosa, Bulimia nervosa, Temperament, Character, Outcome

## Abstract

**Background:**

Eating Disorders (EDs) are complex psychiatric pathologies characterized by moderate to poor response to treatment. Criteria of remission and recovery are not yet well defined. Simultaneously, personality plays a key role among the factors that determine treatment outcome. The aim of the present research is to evaluate the possibility of temperamental and character traits to predict the long-term outcome of ED.

**Method:**

A sample of 25 AN and 28 BN female patients were re-assessed face-to-face after a minimum 5-years-follow-up through SCID-I, EDI-2 and TCI-R. Regression Analyses were performed to ascertain the possibility of TCI-R dimensions at the first visit to predict the long-term outcome.

**Results:**

Clinical and psychopathological symptoms significantly decreased over the time and 23% of participants no longer received a categorical ED diagnosis after at least 5 years of follow-up. TCI-R dimensions failed to predict the absence of a DSM-IV-TR diagnosis in the long term, but Novelty Seeking, Harm Avoidance and Reward Dependence demonstrated to predict the clinical improvement of several EDI-2 scales.

**Conclusions:**

Our results support the idea that temperamental dimensions are relevant to the long-term improvement of clinical variables of ED. Low Novelty Seeking is the strongest predictor of poor outcome.

## Background

Eating Disorders (EDs) are complex psychiatric pathologies characterized by physical, behavioral, emotional and cognitive symptoms with moderate to poor long-term outcome after treatment [1-3].

Multiple factors contribute to recovery and remission in EDs, and these concepts are not clearly and universally accepted at the present time. One of the problems is that the outcome is evaluated in the first place by using physical and behavioral symptoms while the other two symptoms (emotional and cognitive) are not taken into account. The latter symptoms however are the factors that lead to the interpersonal and psychosocial disabilities, most commonly observed in not fully recovered ED patients over time.

Another reason for the lack of a clear definition of outcome is the high rate of Axis II comorbidity [4,5] in particular with Cluster B and C personality disorders.

Distinctive temperament and character profiles have been described according to Cloninger’s [6] psychobiological model that precede the onset of ED and remain quite stable after the remission [7-9]: patients with Anorexia Nervosa (AN) usually have low Novelty Seeking and Cooperativeness and high Persistence; patients with Bulimia Nervosa (BN) tend to show high Novelty Seeking; and both, AN and BN, share high Harm Avoidance and low Self-Directedness.

Clinicians observe the overlap between the core symptoms of EDs, characteristic personality traits and other psychiatric comorbidities. Accordingly, the central role of personality has been highlighted when a unitary conception of EDs and comorbidities beyond the co-diagnosis was attempted [10].

Therefore, we hypothesize that personality dimensions, especially temperamental dimensions, might be a useful tool in the early prediction of the long-term outcome of EDs after treatment. So the aim of the present research is to evaluate whether temperamental and character traits can be used to predict the long-term outcome of Anorexia Nervosa and Bulimia Nervosa.

## Methods

### Participants

All clinical records of female patients diagnosed with AN or BN according to DSM-IV-TR criteria between January 2000 and December 2012 at the outpatient service for Eating Disorders were analyzed. The study was done at the University Policlinic Mater Domini of Catanzaro in Italy. Patients followed an individual therapeutic intervention at the outpatient service for 12 months: once a week for the first six months and biweekly from the seventh until the end. The intervention consisted in nutritional rehabilitation, psycho-education and cognitive restructuring. Cognitive restructuring was intended to identify and correct the cognitive mechanisms that underlie and support the ED. At the end of therapy the follow-up was structured through clinical controls with a psychiatrist and a dietitian, twice a month for six months, once a month for six months and subsequently from two to six times a year for the next three years. During the follow-up period, patients did not undergo any other psychotherapy.

Inclusion criteria: female gender; first diagnosis and treatment for ED at the outpatient unit between January 2000 and December 2007; having completed the Cognitive Behavior Therapy (CBT); having completed an eating educational program for at least a year, and periodical follow-up controls at the clinic for at least 5 years. Patients matching the criteria were contacted by telephone. Patients were informed of the aim of the study, that their participation was voluntary and that their personal data would be kept confidential; they were invited to make a check-up at the clinic, to write their diet diary during the week before the visit and to be evaluated through psychological questionnaires at the clinic. Eighty-six out of one-hundred-eighty-three patients matched the criteria; twenty-one of them could not be reached, so sixty-five were contacted; fifty-three of them gave verbal informed consent to participate in the present research by phone, and a written informed consent (previously authorized by the ethical committee) was given by these women before any assessment when they came to the clinic.

All participants with an initial diagnosis of AN and BN were included as members of a sole sample. This choice finds a justification both on the transdiagnostic model of EDs [11] and the frequent migration across diagnosis in EDs [12,13].

This research protocol was finally approved by the ethics committee of the Azienda Ospedaliera Universitaria Mater Domini of Catanzaro on February 12th 2012 according to local and international standards.

### Assessments

Diagnosis of EDs according to DSM-IV TR [14], was made both at the first access to the outpatient clinic (t0) and during the last evaluation (t1) by means of SCID-I [15], dietary diaries and BMI.

At t1, two psychiatrists with adequate training in the field of EDs, and who did not have any previous contact with participants, interviewed them face to face through the SCID-I. Additional information regarding age, marital status, education, occupation, eating behavior, purging behavior, type and frequency of physical activity was also collected. In order to ascertain if disordered eating behaviors were or were not still present, participants’ diet diaries were reviewed by the dietitian of the outpatient service. The dietitian also measured them wearing light indoor clothing and no shoes using a stadiometer (Seca 220, GmbH & Co., Hamburg, Germany) and a balance scale (Seca 761, GmbH & Co., Hamburg, Germany). Then individual’s Body Mass Index (BMI, kg/m^2^) was calculated.

Participants were subsequently invited to answer the same tests they had already responded to at t0: the Eating Disorder Inventory2 (EDI-2) and the Temperament and Character Inventory Revised (TCI-R).

The EDI-2 [16] is a worldwide validated questionnaire that provides a multidimensional evaluation of the very remarkable psychological characteristics of AN and BN through eleven subscales: Drive for Thinness (DT), Bulimia (BU), Interoceptive Awareness (IA), Asceticism, Body Dissatisfaction (BD), Perfectionism (P), Interpersonal Distrust, Impulse Regulation (IR), Ineffectiveness (IN), Maturity Fears (MF), and Social Insecurity (SI). In the literature [17], high Cronbach’s alphas (range: 0.80–0.91) were reported for the internal consistency of the EDI-2 scales and high test–retest reliability in patients with ED diagnoses (range: 0.81–0.89) [18].

The TCI-R [19,20] is a questionnaire based on Cloninger’s neurobiological personality theory [6] that assesses personality on 4 temperamental (Novelty Seeking, NS; Harm Avoidance, HA; Reward Dependence, RD; Persistence, P) and 3 character dimensions (Self-directedness, SD; Cooperativeness, C; Self-transcendence, ST) that can be further divided into 29 scales. The questionnaire has been translated into many languages, validated in different countries and worldwide applied both in clinics and research.

### Statistical design

Statistical Packages for the Social Sciences (SPSS) version 18 was used for statistical analysis. Data are presented as means, standard deviations (SD), frequencies and percentages.

Delta percentage of changes (∆) between t0 and t1were calculated through the following formula: *Δ* = (tl score - t0 score/t0 score) × 100; where positive result indicate an increment of the score at t1 and vice versa.

Univariate analysis included t-Test for independent samples comparison; Paired-Samples t-Test for numerical data (Table 1) and Chi-square for categorical ones were used to evaluate the change between t0 and t1 in EDI-2 scales and TCI-R dimensions.

**Table 1:** Paired sample t-test of EDI-2 and TCI-R scores between t0 and t1

		**t0**		**t1**		**Paired sample t-test**	
		**Mean**	**SD**	**Mean**	**SD**	**t**	**P**
EDI-2	DT	14,4	6,9	7,5	7,2	3,623	**0,002
	BU	6,4	7,2	2,9	4,7	2,537	*0,02
	BD	15,4	7,6	14,0	8,2	0,73	0,474
	IA	12,6	7,9	8,5	7,5	2,455	*0,024
	ASC	8,4	4,8	5,9	3,9	1,985	0,062
	P	5,5	4,9	5,4	4,3	0,048	0,963
	MF	8,6	5,8	10,3	7,5	-0,977	0,341
	IR	10,3	6,2	6,7	7,1	1,872	0,077
	IN	12,6	8,4	11,5	8,9	0,629	0,537
	SI	10,3	5,5	9,0	9,3	0,587	0,564
	ID	7,3	4,8	5,5	5,2	1,19	0,249
TCI-R	NS	92,3	20,5	105,1	14,2	-2,621	*0,019
	HA	125,0	16,4	110,2	16,8	3,999	***0,001
	RD	99,2	15,8	100,6	11,8	-0,305	0,764
	PERS	103,2	30,4	112,5	14,9	-1,309	0,209
	SD	98,6	22,1	125,8	22,5	-4,81	***0,001
	C	130,6	19,9	132,7	12,7	-0,379	0,71
	ST	58,9	14,0	61,7	17,1	-0,63	0,538

DT: Drive for Thinness; BU: Bulimia; BD: Body Dissatisfaction; IA: Interoceptive Awareness; ASC: Asceticism; P: Perfectionism; MF: Maturity Fears; IR: Impulse Regulation; IN: Ineffectiveness; SI: Social Insecurity; ID: Interpersonal Distrust; NS: Novelty Seeking; HA: Harm Avoidance; RD: Reward Dependence; P: Persistence; SD: Self-directedness; C: Cooperativeness; ST: Self-transcendence.

***Bold character***: significant differences. *p < .05; **p < 0.1; ***p < .001.

Multivariate analysis included both Forward Stepwise Multiple Logistic Regression Analysis and Forward Stepwise Multiple Linear Regression Analysis. The first was performed to assess the probability of predicting the presence of an ED diagnosis according to DSM-IV-R at t1 (ED diagnosis during the last 12 months = 1; absence of ED diagnosis during the last 12 months = 0) considering TCI-R dimensions at t0 (NS, HA, RD, P, SD, C, ST) as independent predictors (continuous) corrected by age and duration of follow-up. Then a series of Multiple Linear Regression Analyses were performed where the delta of the eleven scales of EDI-2 were alternatively considered as dependent variables and the seven dimensions of TCI-R at t0 as independent predictors corrected by age and duration of follow-up (Table 2). Probability for stepwise entry and removal were 0.2 and 0.4. Type I error was set at p ≤ 0.05. Odds Ratio (OR) and Cohen’s effect sizes (ESs) were calculated; *ES* ≤ 0.2 from 0.3 to 0.6, from 0.7 to 1.2 and > 1.2 were respectively considered as trivial, small, moderate and large.

**Table 2:** Linear regression analysis

**Dependent variable**	**Predictor**	**Beta**	**t**	**Sig.**
∆BD	NS	-1,085	-4,752	0,000
	HA	-0,668	-3,218	0,007
	RD	0,448	2,33	0,037
∆ASC	NS	-0,775	-3,679	0,003
∆IR	NS	-0,747	-3,013	0,009
∆SI	HA	-0,692	-3,358	0,005
	NS	-0,528	-2,28	0,04

∆: delta t0-t1; BD: Body Dissatisfaction; ASC: Asceticism; IR: Impulse Regulation; SI: Social Insecurity; NS: Novelty Seeking; HA: Harm Avoidance; RD: Reward Dependence.

## Results

### Sample description

Twenty out of 53 participants (38%) had received an initial diagnosis of Anorexia Nervosa Restricting Type (ANR), 5 (9%) of Anorexia Nervosa Binge-Eating/Purging Type (ANP) and 28 (53%) of Bulimia Nervosa Purging Type (BNP) at t0. The average age of the sample was 22.9 ± 7.2 years (AN = 21.4 ± 5.9; BN = 24.4 ± 8.3) and the BMI was 18.8 ± 5.8 (AN = 15.8 ± 2.0; BN = 21.7 ± 3.8). The mean lasting follow-up since the first visit was 8.5 ± 3.3 years.

### Changes t0-t1

**Figure 1:**
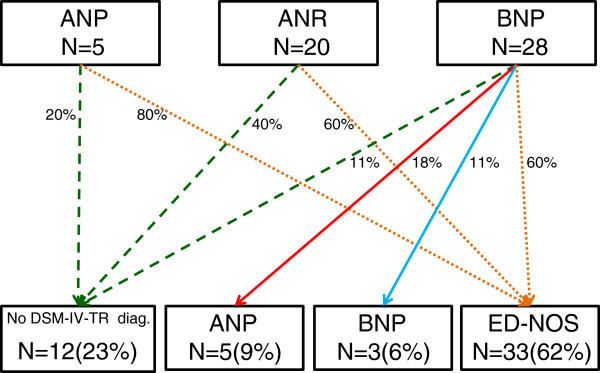
**Diagnostic migration of participants from t0 to t1.** ANP: Anorexia Nervosa Binge-Eating/Purging Type; ANR: Anorexia Nervosa Restricting Type; BNP: Bulimia Nervosa Purging Type; ED-NOS: Eating Disorder Not Otherwise Specified.

Figure 1 illustrates the diagnostic migration of patients between t0 and t1. A high percentage of patients moved from their original diagnosis of ANP, ANR and BNP to ED-NOS (respectively 80%, 60% and 60%) while the percentage of those who did not match the DSM-IV-TR diagnostic criteria for any ED during the prior twelve months was lower (respectively 20%, 40% and 11%). The final distribution of diagnosis according to DSM-IV-TR criteria at t1 were: 23% no diagnosis, 9% ANP; 6% BNP; 62% ED-NOS.

At t1 the mean age was 30.1 ± 7.1 years and the average BMI was 21.1 ± 3.3; the BMI of patients with an initial diagnosis of AN raised to 20.4 ± 2.4 (t = -6.325; p < 0.001) while that of BN decreased to 20.0 ± 4.3(t = 1.533; p = 0.160).

The only significant difference between AN and BN participants at t0 in relation to the scores of TCI-R and EDI-2 regarded the scale Bulimia of EDI-2 (AN = 3.0 ± 3.9 vs BN = 10.2 ± 7.9; t = 4.129; p < 0.001). This result further confirmed the choice of including all patients in the same sample for further statistical analysis.

**Figure 2:**
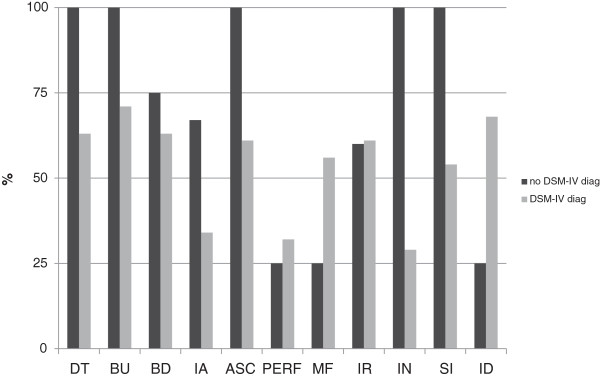
**Percentage of participants with EDI-2 scores within 1SD of normal population.** no DSM-IV diag: patients that no longer matched criteria for an ED diagnosis during the last year; DSM-IV diag: patients with ED criteria for an ED diagnosis during the last year; DT: Drive for Thinness; BU: Bulimia; BD: Body Dissatisfaction; IA: Interoceptive Awareness; ASC: Asceticism; P: Perfectionism; MF: Maturity Fears; IR: Impulse Regulation; IN: Ineffectiveness; SI: Social Insecurity; ID: Interpersonal Distrust.

Table 1 shows the results of the Paired sample t-Test for EDI-2 and TCI-R scores between t0 and t1. With the only exception of Maturity Fears, all the EDI-2 scores decreased at t1. Significant differences were only observed in Drive for Thinness, Bulimia and Interoceptive Awareness. No participant reached EDI-2 scores within 1 standard deviation of normal population values in all the scales at t1. Still all participants with no formal diagnosis of ED at t1 showed DT, BU, IN, ASC and SI scores within normative data while only a small proportion were within normal limits for PERF, ID and MF (Figure 2).

With regards to TCI-R significant changes at t1 were found in three dimensions: Novelty Seeking and Self-Directedness significantly increased while Harm Avoidance significantly decreased.

### Multivariate analysis

Temperamental (NS, HA, RD and P) and character (SD, C and ST) dimensions of TCI-R failed to predict the long-term outcome (absence of DSM-IV-TR diagnosis at t1) as none of them entered among the predictive variables in the Logistic Regression Analysis.

On the other hand, when Multiple Linear Regression Analyses were performed, the delta of several scales of EDI-2 were predicted by some temperamental dimensions at t0 (Table 2).

More in detail, the increase of Body Dissatisfaction (R^2^=,533; F = 5,859; p=,006) appeared to be related to low NS, low HA and high RD at t0. The increase of Asceticism (R^2^=,445; F = 4,402; p=,018) and Impulse Regulation (R^2^=,280; F = 3,693; p=,05) scores appeared to be predictable by low NS at t0. Higher Social Insecurity at t1 was related to low NS and HA at t0 (R^2^=,401; F = 3,825; p=,028). In other words, low NS at t0 predicted the aggravation of BD, ASC, IR and SI scales of EDI-2; low HA predicted higher BD and SI scores at t1; and high RD predicted higher BD at t1.

## Discussion

The current investigation aims to assess the possibility to use temperamental and character dimensions when predicting the long-term outcome of EDs. The first step was to evaluate the outcome after adequate treatment and a long follow-up period.

In agreement with other studies, our results showed an overall reduction of general psychopathology and a considerable proportion of participants who scored within the normal range in several dimensions of the EDI-2. Only twenty-three percent of participants did no longer match an ED diagnosis during the last year whereas a high proportion of patients still fell within the ED-NOS category. An improvement in some personality traits was also observed. Temperament and character dimensions failed to predict the outcome in terms of DSM-IV TR diagnostic criteria, but they were successful in predicting the clinical changes of patients.

There is an ongoing controversy on the criteria to evaluate the outcome of EDs [21-24] that derives from the inner complexity of these disorders. Efforts to define and set solid criteria for response, remission and recovery following treatment have been done [25-27] without reaching a definite international consensus. On the other hand large differences in the rates of remission and recovery emerge across studies as a consequence of different methodological procedures [27,28] and/or different outcome criteria [29,30].

In the present study we used operational criteria to classify patients in the long term. This showed that only a small portion of them had reached a good outcome. In the light of critical reading of current literature, even if the requirements for recovery deduced from the DSM-IV [24,31] are much broader than those used in most studies, we still believe that no longer matching the DSM criteria does not imply to have reached a state of full recovery (i.e. not being ugly does not imply being beautiful). Psychopathological data, changes in the rating scales, data related to personality as well as their relationship could improve not only the validity but also the real utility of the outcome criteria [32]. This consideration is supported by the following facts: first, some features remain symptomatic over time to a subclinical level among patients that do not maintain the ED diagnosis [33]; second, personality dimensions fail to predict if subjects will maintain a DSM-IV diagnosis in the long-term while the same personality dimensions are able to predict some important clinical modifications.

According to literature, ED partial recovery could mainly be defined by the amelioration of physical and behavioral aspects (no longer meeting criteria for an ED, no pathological eating behaviours for 3 months and BMI ≥ 18.5) while the additional normalization of EDE-Q scores would guarantee the full recovery through the recovery of emotional and cognitive symptoms [32]. In the present research we applied the same criteria for a longer period (12 months) using the EDI-2 instead of the EDE-Q. Not only few dimensions showed a significant reduction over time (DT, BU and IA), but it was also verified that no woman had obtained the normalization of all scores of EDI-2 in the long term. This observation is in agreement with the above discussed and emphasizes the poor outcome in the long-term, the problem of treatment-resistance and the risk of relapse.

Patients with ED have pre-morbid and well defined personality traits that influence genesis and maintenance of the disorder [34,35]. Even if some confounding factors should be borne in mind when evaluating the personality of ED patients [4], several studies have demonstrated that the treatment for the ED is, to a certain extent, able to modify personality dimensions. Recovery drives personality traits closer to those of healthy controls [36,37] with an overall reduction of Harm Avoidance and Self-Transcendence and the increase of Reward Dependence, Self-Directedness and Cooperativeness [38-40]; nevertheless in the same way that other symptoms may persist after recovery, patients recovered from AN and BN tend to maintain higher Harm Avoidance and lower Self-Directedness than people who never had an ED [41]. The present research confirmed the same personality changes in the long term with regard to HA and SD but also a significant increase of NS through time. This last peculiar increment may be explained because in our sample, where AN and BN patients were considered together, the largest improvement was obtained by patients with an initial diagnosis of AN-R, typically characterized by low NS scores [7-9].

Agüera et al. [40], in an interesting research but with an opposite reasoning, demonstrated that the delta of EDI-2 total score was a specific clinical predictor of TCI-R changes after treatment. From our point of view, this bidirectional relationship between the psychopathology (epiphenomenon or shallower element) described by EDI-2 and the structure of personality (structural and deeper element) described by TCI, can be read in the sense that visible changes regarding the surface elements reflect changes in depth. So, changes in depth (personality traits) are those that could subsequently predict the more easily detectable psychopathological changes at the clinical observation for their shallower position.

Our aim, in any case, was to test if the personality dimensions initially observed in ED patients were able to predict the clinical long-term outcome. The research failed to predict the outcome by means of the absence or presence of a DSM-IV-TR diagnosis in the long term. Nevertheless, in contrast with a recent research in which personality traits had no significant interaction with recovery [31], the results of regression analysis have demonstrated the important role of temperamental dimensions (NS, HA and RD) as independent predictors of some clinical modifications.

Previous papers have demonstrated that TCI-R dimensions are able to predict some clinical improvement: high HA has predicted favorable clinical changes after a six-month therapy of Brief Adlerian Psychodynamic Psychotherapy for ED patients [42] while low SD and low C have been found to correlate with drop-out from treatment among ED patients [35]. Present data confirm that low HA is related to poor outcome with higher body dissatisfaction and social insecurity in the long term. From this perspective, high HA scores could be considered not only a characteristic feature of EDs but also a beneficial trait. The need of approval related to RD helps to understand why high scores in this dimension are correlated to further higher body dissatisfaction. Despite a significant increase at t1, the mean NS score remained quite stable on average values [20]. Low NS was previously identified as a characteristic of the non-responder group in the short term [43]; our data support this results, in this case predicting the long-term outcome: initial low NS scores were associated to the worsening of relevant clinical symptoms (BD, ASC, IR and SI). This fact leads to reflect on the important role of NS on determining and sustaining the psychopathology of EDs.

The present study has some strengths and limits. The first limit is the reduced sample size; it is due to the narrow criteria of inclusion used to guarantee a long follow-up and also to the fact that data collection was made in an outpatient unit of a small city. On the other hand it can also be considered a strength as all patients were treated by the same therapeutic team with a long average of follow-up.

The second limit could be including patients with AN and BN as members of a sole sample. This methodological choice, previously explained, found further justification with the absence of significant differences between patients except in the BU scale of EDI-2.

The third limit is not having considered comorbidities [44], but the aim of the study was only to evaluate the power of temperament and character dimensions to predict clinical modifications.

A possible forth limit regards that results rely solely on self-report data, particularly self-assessment of personality characteristics; to reduce this limit, reliable and worldwide validated questionnaires were used.

## Conclusions

The early identification of prognostic features has fundamental implications in relation to the goal of treatment, the therapeutical strategies and the health professionals to include in the therapeutic team [24]. As with other psychiatric disorders, ED residual symptoms correlate with increased likelihood of relapse in the long-term. The early identification of the elements that are most likely not to be improved could lead to more targeted and effective therapeutic interventions. Among diagnostic factors, personality is the one that suits to provide prognostic indications because clinical symptoms may vary widely over time in relation to severity and diagnostic migrations, while personality traits, especially temperamental ones, are structural elements.

Our results support that some temperamental dimensions may be useful tool to predict the outcome in the long term, and among them, low Novelty Seeking could be the strongest predictor of non-response.

## Abbreviations

ED: Eating disorders; CBT: Cognitive behavioural therapy; AN-R: Anorexia nervosa-restricting type; AN-P: Anorexia nervosa-purging type; BN-P: Bulimia nervosa-purging type; ED-NOS: Eating disorder not otherwise specified; DSM-IV-TR: Diagnostic and statistical manual of mental disorders iv text revised; EDI-2: Eating disorders inventory-2; DT: Drive for thinness; BU: Bulimia; BD: Body dissatisfaction; IA: Interoceptive awareness; ASC: Asceticism; P: Perfectionism; MF: Maturity fears; IR: Impulse regulation; IN: Ineffectiveness; SI: Social insecurity; ID: Interpersonal distrust; TCI-R: Temperament and character inventory revised; NS: Novelty seeking; HA: Harm avoidance; RD: Reward dependence; P: Persistence; SD: Self-directedness; C: Cooperativeness; ST: Self-transcendence.

## Competing interests

All authors declare that they have no conflicts of interests.

## Authors’ contributions

CSG designed the study. DC and FP collected the patient data. CSG performed the statistical analyses. CSG and PDF wrote the first draft of the manuscript. All authors commented on and approved the final manuscript.

## Pre-publication history

The pre-publication history for this paper can be accessed here:

http://www.biomedcentral.com/1471-244X/13/288/prepub
